# Gradient of Expression of Dopamine D2 Receptors Along the Dorso-Ventral Axis of the Hippocampus

**DOI:** 10.3389/fnsyn.2019.00028

**Published:** 2019-10-15

**Authors:** Valentyna Dubovyk, Denise Manahan-Vaughan

**Affiliations:** ^1^Medical Faculty, Department of Neurophysiology, Ruhr University Bochum, Bochum, Germany; ^2^International Graduate School of Neuroscience, Ruhr University Bochum, Bochum, Germany

**Keywords:** dopamine receptor, hippocampus, immunohistochemistry, rodent, dorso-ventral axis

## Abstract

Dopamine D2-like receptors (D2R) play an important role in the regulation of hippocampal neuronal excitability and contribute to the regulation of synaptic plasticity, the encoding of hippocampus-dependent memories and the regulation of affective state. In line with this, D2R are targeted in the treatment of psychosis and affective disorders. It has been proposed that the dorso-ventral axis of the hippocampus can be functionally delineated into the dorsal pole that predominantly processes spatial information and the ventral pole that mainly addresses hippocampal processing of emotional and affective state. Although dopaminergic control of hippocampal information processing has been the focus of a multitude of studies, very little is known about the precise distribution of D2R both within anatomically defined sublayers of the hippocampus and along its dorsoventral axis, that could in turn yield insights as to the functional significance of this receptor in supporting hippocampal processing of spatial and affective information. Here, we used an immunohistochemical approach to precisely scrutinize the protein expression of D2R both within the cellular and dendritic layers of the hippocampal subfields, and along the dorso-ventral hippocampal axis. In general, we detected significantly higher levels of protein expression of D2R in the ventral, compared to the dorsal poles with regard to the CA1, CA2, CA3 and dentate gyrus (DG) regions. Effects were very consistent: the molecular layer, granule cell layer and polymorphic layer of the DG exhibited higher D2R levels in the ventral compared to dorsal hippocampus. D2R levels were also significantly higher in the ventral Stratum oriens, Stratum radiatum, and Stratum lacunosum-moleculare layers of the CA1 and CA3 regions. The apical dendrites of the ventral CA2 region also exhibited higher D2R expression compared to the dorsal pole. Taken together, our study suggests that the higher D2R expression levels of the ventral hippocampus may contribute to reported gradients in the degree of expression of synaptic plasticity along the dorso-ventral hippocampal axis, and may support behavioral information processing by the ventral hippocampus.

## Introduction

Synaptic plasticity and memory formation in the hippocampus, in their essence, are dependent on excitatory glutamatergic transmission (Citri and Malenka, 2008; Lisman, 2017). However, various neurotransmitter systems exert a modulatory role, thereby influencing the direction of change (Kemp and Manahan-Vaughan, 2005), effectiveness (Hansen and Manahan-Vaughan, 2015), duration (Twarkowski and Manahan-Vaughan, 2016) or robustness (Manahan-Vaughan and Kulla, 2003) of synaptic strength, and learning and memory. The dopaminergic system is one such modulatory influence on neuronal and synaptic activity (Kulla and Manahan-Vaughan, 2000; Lemon and Manahan-Vaughan, 2012; Hansen and Manahan-Vaughan, 2014; Madadi Asl et al., 2019).

Dopamine exerts its action by means of slow modulation of fast neurotransmission through its G-protein-coupled receptors. Five types of dopaminergic receptors have been identified that are subdivided into D1 (D1 and D5) and D2 (D2, D3 and D4) classes of receptors based on their ability to modulate cAMP production and their structural, biochemical and pharmacological properties (Sibley and Monsma, 1992; Vallone et al., 2000; Beaulieu and Gainetdinov, 2011). While the D1-class receptors stimulate adenylyl cyclase (AC) activity and therefore cAMP production, the D2-class receptors inhibit AC activity, thus hindering cAMP production. D2 receptors also modulate the beta-arrestin/GSK3 pathway and can alter the excitability of hippocampal mossy cells through this mechanism (Etter and Krezel, 2014). Both classes of dopamine receptors are postsynaptically expressed on dopamine-receptive cells (Rankin et al., 2009). Interestingly, only the D2-class receptors exist in different isoforms (Gingrich and Caron, 1993). The D2 dopamine receptor (D2R) exists in D2S (short) and D2L (long) variants. Whereas D2S is predominantly expressed presynaptically, D2L is mostly postsynaptically expressed (Usiello et al., 2000; De Mei et al., 2009). Thus, the D2R enables a more complex modulation of neuronal circuitry than that exerted by other dopamine receptor types. Moreover, the D2R is the only dopamine receptor that has been directly implicated in several brain conditions, such as drug addiction, Parkinson’s disease and schizophrenia (Wong et al., 1986; Volkow et al., 2007; Chaudhuri and Schapira, 2009). Therefore, in-depth knowledge about the spatial expression pattern of D2R would be beneficial for understanding not only the physiological role, but also, the pathological role of this receptor.

Surprisingly, despite the wealth of studies on the dopaminergic system, very little is known about D2R expression in the hippocampus proper (Gasbarri et al., 1997; Beaulieu and Gainetdinov, 2011); most studies to date, focused on the basal ganglia and occasionally on the frontal cortex (Levey et al., 1993; Yung et al., 1995; Santana et al., 2009; Lavian et al., 2018). The most extensive examination of D2R expression in rat hippocampus up to now was performed by Yu et al. (2019). In their work they showed that D2R is mainly expressed in cells of the Stratum pyramidale (sp) and Stratum radiatum (sr) layers of the CA1–3 regions, in the dentate gyrus (DG), and in axon terminals innervating the Stratum lacunosum-moleculare (slm) of the CA1 of the dorsal hippocampus. Despite their extensive investigation, the authors omitted scrutiny of the overall expression level of D2R across the layers of the trisynaptic circuit of the hippocampus, as well as across its dorso-ventral axis.

The hippocampal dorso-ventral axis is functionally segregated into the dorsal part that is essential for visuo-spatial information processing, the ventral part that is involved in emotional, motivational and affective responses, and the intermediate part that integrates information from both poles (Bast et al., 2009; Fanselow and Dong, 2010; Strange et al., 2014). Hippocampal dopamine originates from neurons of the ventral tegmental area (VTA) and locus coeruleus (LC; Lisman and Otmakhova, 2001; Lemon et al., 2009; Smith and Greene, 2012). Projections from the LC predominantly terminate in the dorsal hippocampus (Kempadoo et al., 2016), whereas projections from the VTA are the strongest in the ventro-intermediate hippocampal two-thirds (Gasbarri et al., 1994). This segregation in innervation by dopamine-containing terminals (Duszkiewicz et al., 2019) further suggests that dopaminergic transmission may exert a non-uniform influence across the hippocampal longitudinal axis. This may be mediated by the degree of dopamine release, but also by differences in receptor expression. Here, the D2R is of particular interest: it is intrinsically involved in the regulation of hippocampal basal synaptic transmission and of the robustness of synaptic plasticity (Manahan-Vaughan and Kulla, 2003).

In this work, we used an immunohistochemical approach to conduct a detailed study of the expression and distribution pattern of D2R across the layers of the DG and cornu ammonis (CA) regions of the dorsal, intermediate and ventral hippocampus of the rat. We observed differences in the expression gradient of this receptor along the dorso-ventral hippocampal axis, and within the microcircuitry of the hippocampus. Our data provide new insights into hippocampal D2R expression that may help in understanding the cellular mechanisms behind D2R modulation of neuronal excitability and synaptic plasticity in the hippocampus.

## Materials and Methods

### Animals

All experiments were conducted using 8–12-week-old male Wistar rats (Charles River Laboratories, Sulzfeld, Germany). Animals were housed in custom-made climatized and ventilated holding cupboards in an animal-housing room with a controlled 12-h light/dark cycle. No female rats were housed in the room. Water and food were available *ad libitum*. The study was carried out in accordance with the European Communities Council Directive of September 22nd, 2010 (2010/63/EU) for care of laboratory animals and all experiments were conducted according to the guidelines of the German Animal Protection Law. They were approved in advance by the North Rhine-Westphalia (NRW) State Authority (Landesamt für Arbeitsschutz, Naturschutz, Umweltschutz und Verbraucherschutz, NRW). All efforts were made to reduce the number of animals used.

### Slice Preparation

Wistar rats were deeply anesthetized with sodium pentobarbital and transcardially perfused with cold Ringer’s solution + heparin (0.2%) followed by 4% paraformaldehyde (PFA) in phosphate buffered saline (PBS, 0.025 M). Brains were removed, fixed in 4% PFA for 24 h, and cryoprotected in 30% sucrose in 0.1 M PBS for at least 3 days. Serial 30-μm thick horizontal sections were collected with a freezing microtome. For each animal (*N* = 10), three horizontal sections from the most dorsal (between 3.6 and 4.1 mm posterior to Bregma), middle intermediate (around 5.6 mm posterior to Bregma) and most ventral hippocampal parts (between 7.1 and 7.6 mm posterior to Bregma) were simultaneously used for immunohistochemical staining (Figure 2).

**Figure 1:**
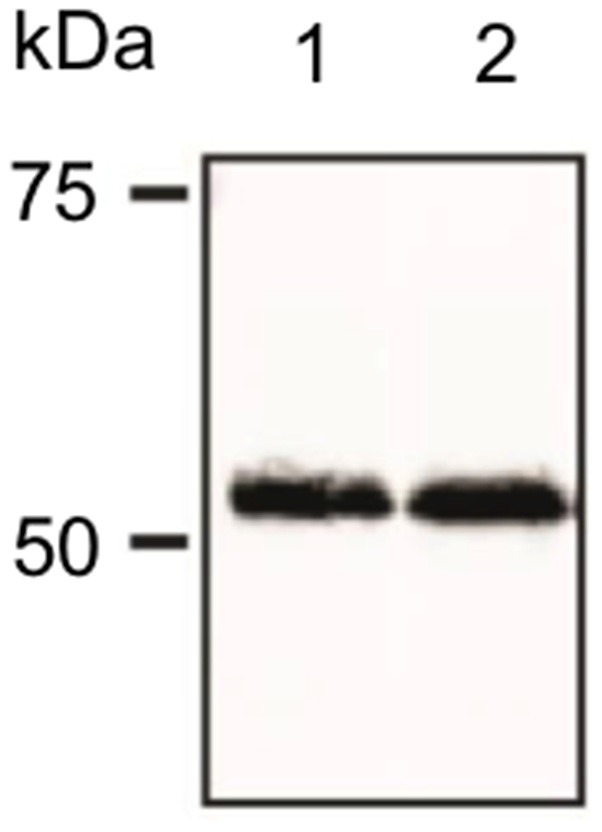
Verification of antibody specificity. Example of a western blot showing binding specificity of the dopamine D2-like receptor (D2R) antibody used in the immunohistochemistry experiments. The antibody labeled a band of ca. 55 kDa, corresponding to the reported kDa weight of the target receptor as reported by others (Gemechu et al., 2018).

**Figure 2:**
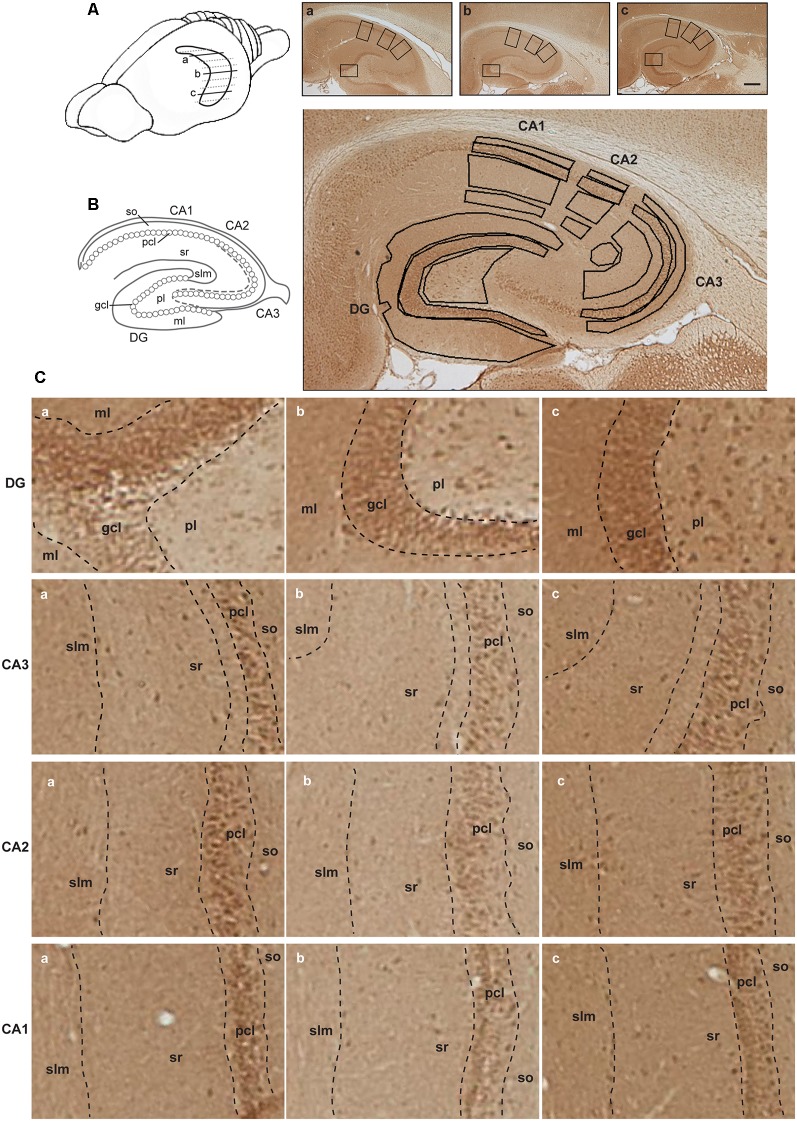
Illustration of hippocampal separation into longitudinal and transverse axes. **(A)** A drawing of the rat brain with horizontally sectioned hippocampus is presented. Representative images of immunohistochemically stained sections against D2R of the dorsal (a), intermediate (b) and ventral hippocampus (c) from the same animal are shown. Scale bar is 500 μm. Squares on each image correspond to zoom-in fragments on the panel **(C)**. **(B)** Schematic representation of the hippocampal transverse axis and laminar separation (left). Immunohistochemically stained section with marked regions taken for the analysis is depicted (right). Abbreviations correspond to: ml, molecular layer of the DG; gcl, granule cell layer of the DG; pl, polymorphic layer of the DG; so, Stratum oriens of CA3/CA2/CA1; pcl, pyramidal cell layer of CA3/CA2/CA1; sr, Stratum radiatum of CA3/CA2/CA1; and slm, Stratum lacunosum-moleculare of CA3/CA2/CA1. **(C)** A closer view of subsections of the dentate gyrus (DG) and CA regions from the dorsal (a), intermediate (b) and ventral (c) hippocampal sections, including the laminar delineation, is shown.

### Verification of Antibody Specificity

The specificity of the D2R antibody was conducted using western blotting (Figure 1). Following brain removal, the hippocampus was rapidly isolated and then homogenized in 20 mM Tris–HCl buffer (pH 7.4) containing 10% sucrose, followed by centrifugation at 14,000 rpm for 30 min at 4°C in 20 mM Tris–HCl buffer supplemented with protease inhibitor. The supernatant was removed and the pellet was resuspended in 20 mM Tris–HCl buffer supplemented with protease inhibitor. The protein concentration of the samples was determined with a Bradford assay. Protein samples were separated in 8% SDS polyacrylamide gels that were prepared 1 day before use. Prior to gel electrophoresis, the required amount of sample (minimum 10 μg of protein per sample) was mixed with the same amount of twice-concentrated (2×) Laemmli buffer. Samples were then heated for 5 min at 100°C, briefly centrifuged and loaded on to gels. The first line on the gel was always filled with protein marker in the amount of 5 μg, which was then separated into fragments of distinct molecular weights. Gel-electrophoresis was performed for ~1.5 h at 400 V and 15 mA settings per gel. Afterward, gels were removed from the running chamber and left in the cold transfer buffer, while the polyvinyl difluoride membranes (PVDF) were incubated with methanol for approx. 5 min. Membranes were then briefly washed with distilled water and covered with transfer buffer. After the transfer “sandwiches” were assembled, they were placed into the transfer chamber, covered with cold transfer buffer and the wet-transfer of proteins from gel onto membranes began (400 V, 300 mA, 1.5 h).

Membranes were then blocked for 1 h [5% non-fat dry milk, 0.1% Tween 20 in tris-buffered saline (TBS)] at room temperature. Blots were then incubated overnight at 4°C with the primary D2R antibody that was used for immunohistochemistry: rabbit polyclonal anti-D2R (ab-1558, Millipore) at 1:1,000 dilution. The primary antibody was dissolved in boost signal solution (Calbiochem). Membranes were then washed 3 times in 0.1% Tween 20 in TBS and incubated with secondary antibody dissolved in boost signal solution (Calbiochem). Protein bands were visualized using an enhanced chemiluminescence reagent. A distinct band was found at ca. 55 kDa (Figure 1) consistent with specific labeliing of the D2L receptor that is postsynaptically localized, by the antibody used here (Gemechu et al., 2018).

### Immunohistochemistry

Endogenous peroxidase was blocked by pretreating the free-floating brain sections in 0.3% H_2_O_2_ for 20 min. They were then rinsed in PBS and incubated with blocking solution containing 10% normal serum and 20% avidin in PBS with 0.2% Triton X-100 (PBS-Tx) for 90 min at room temperature. Sections were incubated overnight at room temperature with primary D2R antibodies (rabbit polyclonal, 1:250; ab-1558, Millipore) in medium containing 1% normal serum in 0.2% PBS-Tx + 20% biotin. Sections were then rinsed in PBS and incubated with biotinylated goat anti-rabbit (1:500; BA-1,000, Vector) antibodies in 1% normal serum in 0.1% PBS-Tx for 90 min at RT. Afterward, sections were washed in PBS and incubated for 90 min at RT with ABC kit (PK-6,100, Vector) in 1% normal serum in 0.1% PBS-Tx. Finally, sections were washed in PBS and treated with diaminobenzidine (DAB) and 0.01% H_2_O_2_ for approx. 10 min.

### Quantitative Analysis

Regions of interest were defined using the rat brain atlas of Paxinos and Watson (1982) and Nissl staining where every 12th section throughout the whole hippocampus was stained with 0.1% Cresylviolet (c5042, Sigma) as a reference. Fifteen areas of interest included: the molecular layer (ml) of the DG; the granule cell layer (gcl) of the DG; the polymorphic layer (pl) of the DG; the Stratum oriens (so) of CA3/CA2/CA1; the pyramidal cell layer (pcl) of CA3/CA2/CA1; the Stratum radiatum (sr) of CA3/CA2/CA1; and the Stratum lacunosum-moleculare (slm) of CA3/CA2/CA1 on sections taken from the dorsal, intermediate and ventral hippocampal subdivisions (Figure 2). Both, right and left hippocampi were used for the analysis and considered as replicates. For background subtraction, we used receptor-devoid tissue. In the dorsal sections this comprised the fimbria. In the intermediate sections, this comprised the superior thalamic radiation, and in the ventral sections, this was the internal capsule. Pictures of stained sections were acquired with a light microscope (Leica DMR, Germany), equipped with a digital camera (MBF Bioscience) and stored in TIFF format. The regions of interest were analyzed at 2.5× lens magnification. The digitized high-resolution pictures were obtained using Neurolucida software (MBF Bioscience) and quantified using open-source ImageJ software (National Institute of Health). Given that images were acquired with a RGB camera the “Color Deconvolution” plugin in ImageJ was used to deconvolve the color information as well as to convert images to 8-bit format, thus increasing the dynamic range of the signal. R software was used to scale data from several independent stainings/plates using generalized residual sum of squares algorithm to account for batch variability in staining intensities (Kreutz et al., 2007; von der Heyde et al., 2014).

### Statistical Analysis

Receptor expression differences between hippocampal subfields for each layer of the trisynaptic circuit were statistically analyzed using a one-way analysis of variance (ANOVA) followed by Tukey HSD *post hoc* test. Here, statistical comparisons were made for a hippocampal layer of interest between the dorsal, intermediate and ventral subdivisions of 10 animals. In the subsequent text descriptions, “M” corresponds to the mean, while “SD” refers to the standard deviation. All significant differences were defined as *p* < 0.05, *p* < 0.01 or *p* < 0.001. Values are expressed as mean values ± the standard error of the mean (SEM).

## Results

### In the Dentate Gyrus the Dorsal Component Exhibits the Lowest D2R Expression

**Figure 3:**
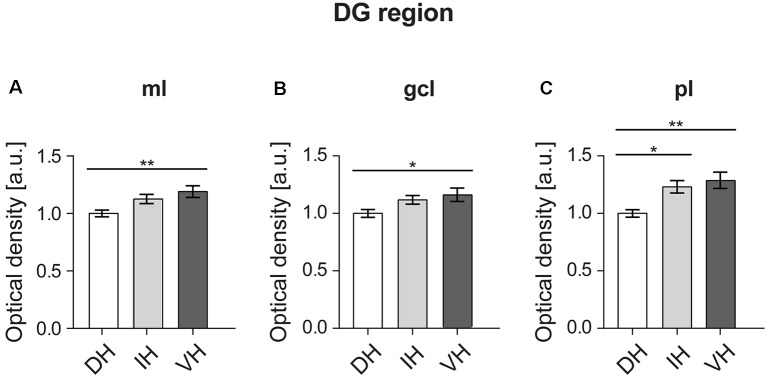
In the DG, the dorsal component exhibits the lowest D2R expression. The molecular layer (ml) **(A)** and granule cell layer (gcl) of the ventral DG **(B)** exhibit higher D2R levels compared to the dorsal DG. The polymorphic layer (pl) **(C)** of the dorsal DG shows the lowest receptor expression as opposed to the ventro-intermediate DG. Values expressed in arbitrary units (a.u.). Error bars indicate standard error of the mean (SEM). *p < 0.05 or **p < 0.01. ml, molecular layer; gcl, granule cell layer; pl, polymorphic layer.

In order to better understand the functional differences across the hippocampal longitudinal axis, we examined the protein levels of D2R in the DG, CA3, CA2 and CA1 regions of the rat dorsal, intermediate and ventral hippocampus by immunohistochemical staining. Of all regions examined, the dorsal DG exhibited the lowest levels of D2R compared to expression levels in the ventral hippocampus. Effects were significant for the molecular layer, granule cell layer and the polymorphic layer (Figure 3).

A one-way ANOVA analysis revealed a significant effect for the three hippocampal parts in the molecular layer of the DG (Figure 3A; *F*_(2,57)_ = 5.665, *p* < 0.01). *Post hoc* Tukey HSD test revealed that the mean score for the dorsal part (*M* = 1, *SD* = 0.12) was significantly lower from the ventral part (*M* = 1.19, *SD* = 0.22). The intermediate part (*M* = 1.12, *SD* = 0.17) did not differ from the other two hippocampal poles (Figure 3A).

Similarly, there was a significant effect in the granule cell layer of the DG (Figure 3B; *F*_(2,57)_ = 3.52, *p* < 0.05) analysis. Again, the *post hoc* test showed a difference in the mean score between the dorsal part (*M* = 1, *SD* = 0.14) and the ventral one (*M* = 1.16, *SD* = 0.26), while the intermediate part (*M* = 1.11, *SD* = 0.16) was not different compared to the other two (Figure 3B).

In case of the polymorphic layer of the DG (Figure 3C), the dorsal part (*M* = 1, *SD* = 0.14) displayed significantly lower D2R levels (*F*_(2,57)_ = 7.691, *p* < 0.01) compared to both, the intermediate (*M* = 1.23, *SD* = 0.24) and the ventral hippocampi (*M* = 1.28, *SD* = 0.32). Receptor expression did not differ between the intermediate and ventral parts (Figure 3C).

### In the CA3 Region, the Ventral Component Shows the Highest D2R Expression

**Figure 4:**
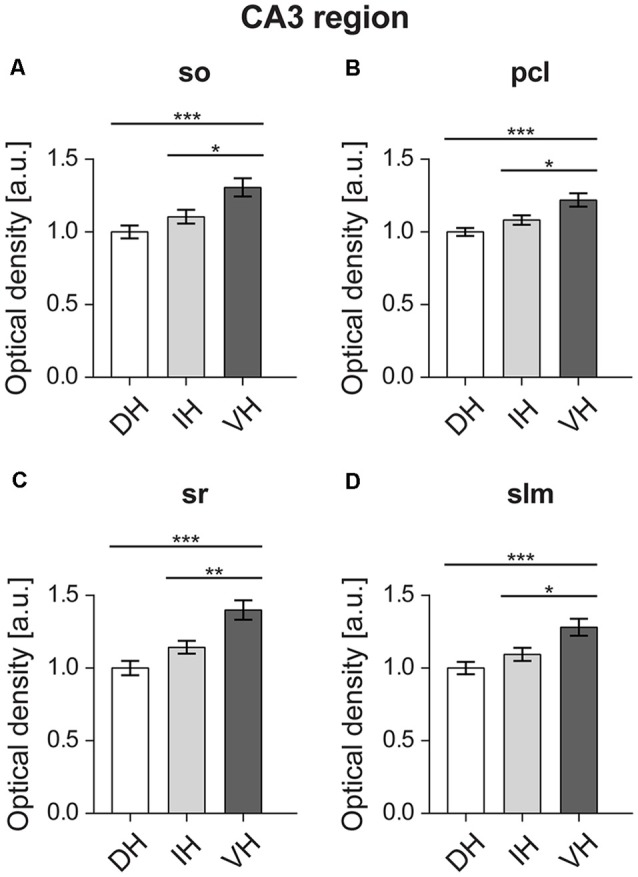
In the CA3 region, the ventral component shows the highest D2R expression. All layers of the ventral CA3 region, namely the Stratum oriens (so) **(A)**, pyramidal cell layer (pcl) **(B)**, Stratum radiatum (sr) **(C)**, and Stratum lacunosum-moleculare (slm) **(D)** display high D2R levels compared to the dorso-intermediate CA3. Values expressed in arbitrary units (a.u.). Error bars indicate SEM. *p < 0.05, **p < 0.01, ***p < 0.001. so, Stratum oriens; pcl, pyramidal cell layer; sr, Stratum radiatum; and slm, Stratum lacunosum-moleculare.

Analysis of the D2R expression between the dorsal, intermediate and ventral hippocampal parts for the four layers of the CA3 region (Figure 4) revealed uniformly higher D2R levels in the ventral third, compared to the dorso-intermediate two-thirds.

Specifically, in the Stratum oriens of the CA3 statistically significant higher D2R expression was found in the ventral CA3 (*F*_(2,57)_ = 8.973, *p* < 0.001). This was apparent between the ventral (*M* = 1.3, *SD* = 0.28) vs. the dorsal part (*M* = 1, *SD* = 0.19), and the ventral vs. the intermediate part (*M* = 1.1, *SD* = 0.21; Figure 4A). In the pyramidal cell layer of the CA3, the ventral part (*M* = 1.21, *SD* = 0.2) also showed significantly higher D2R expression (*F*_(2,57)_ = 9.386, *p* < 0.001) compared to the dorsal layer (*M* = 1, *SD* = 0.12), as well as compared to the intermediate layer (*M* = 1.08, *SD* = 1.44; Figure 4B).

Furthermore, D2R expression was higher in the Stratum radiatum of the ventral CA3 region: the dorsal (*M* = 1, *SD* = 0.21) and intermediate (*M* = 1.14, *SD* = 0.19) parts express significantly less D2R (*F*_(2,57)_ = 14.05, *p* < 0.001) compared to the ventral hippocampus (*M* = 1.39, *SD* = 0.29; Figure 4C). In addition, the Stratum lacunosum-moleculare of the CA3 region followed the same pattern with D2R expression being higher in the ventral component (*F*_(2,57)_ = 8.499, *p* < 0.001; ventral portion: *M* = 1.28, *SD* = 0.26; dorsal: *M* = 1, *SD* = 0.18; and intermediate: *M* = 1.09, *SD* = 0.2; Figure 4D).

### In the CA2 Region, D2R Expression Is Highest in the Ventral Apical Dendrites

**Figure 5:**
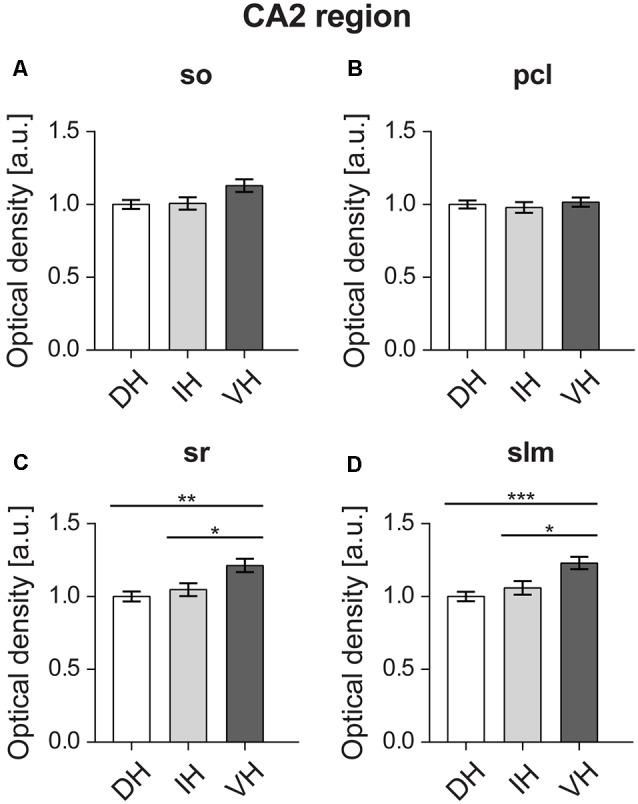
In the CA2 region, D2R expression is highest in the ventral apical dendrites. The D2R is equally expressed in the Stratum oriens (so) **(A)** and pyramidal cell layer (pcl) **(B)** across the longitudinal axis of the CA2 region. Receptor protein expression is highest in the ventral Stratum radiatum (sr) **(C)** and Stratum lacunosum-moleculare (slm) **(D)** as opposed to dorso-intermediate CA2. Values expressed in arbitrary units (a.u.). Error bars indicate SEM. *p < 0.05, **p < 0.01, ***p < 0.001. so, Stratum oriens; pcl, pyramidal cell layer; sr, Stratum radiatum; and slm, Stratum lacunosum-moleculare.

Statistical analysis did not reveal any differences between the dorsal (*M* = 1, *SD* = 0.14), intermediate (*M* = 1, *SD* = 0.19) and ventral parts (*M* = 1.12, *SD* = 0.19) of the Stratum oriens of the CA2 region (*F*_(2,57)_ = 3.324, *p* < 0.05; Figure 5A). Likewise, no differences were found (*F*_(2,57)_ = 0.332, *p* = 0.718) for the pyramidal cell layer of the CA2 region (dorsal: *M* = 1, *SD* = 0.12; intermediate: *M* = 0.97, *SD* = 0.16; ventral: *M* = 1.01, *SD* = 0.13; Figure 5B). However, the ventral Stratum radiatum (*M* = 1.21, *SD* = 0.2) exhibited significantly higher D2R levels (*F*_(2,57)_ = 7.259, *p* < 0.01) as opposed to the dorsal (*M* = 1, *SD* = 0.15) and intermediate parts (*M* = 1.04, *SD* = 0.19; Figure 5C). In case of the Stratum lacunosum-moleculare of the CA2 region, the ventral portion of the hippocampus (*M* = 1.22, *SD* = 0.19) has also exhibited significantly higher expression of D2R (*F*_(2,57)_ = 8.427, *p* < 0.001) compared to the dorsal (*M* = 1, *SD* = 0.14) and intermediate parts (*M* = 1.05, *SD* = 0.2; Figure 5D).

### D2R Expression Is Higher in the Ventral CA1 Region

**Figure 6:**
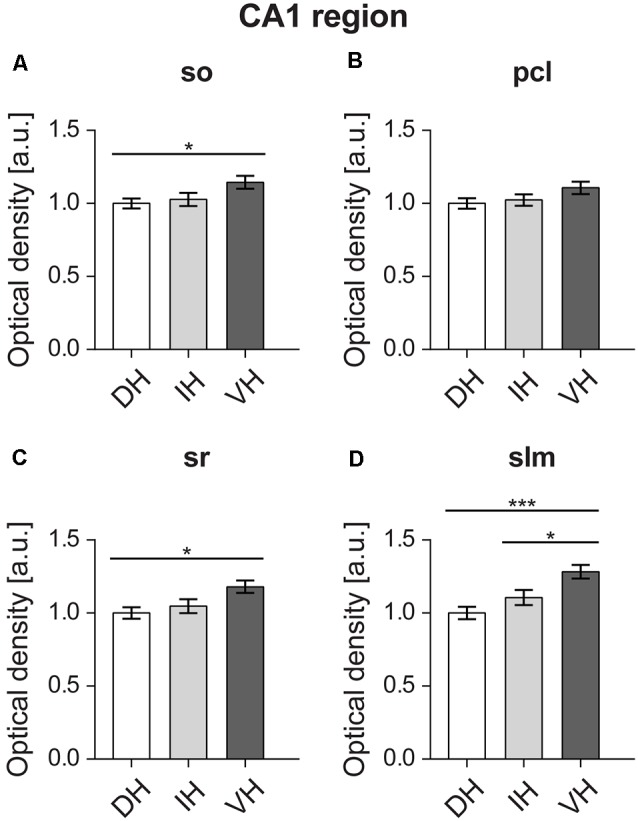
D2R expression is higher in the ventral CA1 region. Proximal dendrites **(A,C)** of the ventral relative to the dorsal CA1 display high D2R protein levels. D2R show similar expression levels throughout the pyramidal cell layer (pcl) layer of the CA1 region **(B)**. Distal dendrites of the ventral CA1 express higher D2R levels compared to the dorso-intermediate parts. **(D)** A gradient of expression of D2R occurs in the Stratum lacunosum moleculare. Expression is highest in the ventral, and lowest in the dorsal SLM. Values expressed in arbitrary units (a.u.). Error bars indicate SEM. *p < 0.05 or ***p < 0.001. so, Stratum oriens; pcl, pyramidal cell layer; sr, Stratum radiatum; and slm, Stratum lacunosum-moleculare.

D2R expression levels were uniformly higher across the non-somatic sublayers of the ventral CA1 region compared to the dorsal CA1 (Figure 6). Thus, expression was found to be the highest in the ventral Stratum oriens (*M* = 1.14, *SD* = 0.19) of the CA1 region (*F*_(2,57)_ = 3.492, *p* < 0.05) as opposed to the dorsal part (*M* = 1, *SD* = 0.14), while no differences were observed with the intermediate part (*M* = 1.02, *SD* = 0.2; Figure 6A). In case of the Stratum radiatum, the ventral hippocampus (*M* = 1.17, *SD* = 0.19) showed significantly higher D2R levels (*F*_(2,57)_ = 4.561, *p* < 0.05) compared to the dorsal part (*M* = 1, *SD* = 0.17). The intermediate sr (*M* = 1.04, *SD* = 0.21) was not significantly different from its counterparts (Figure 6C). The Stratum lacunosum-moleculare followed a similar pattern (*F*_(2,57)_ = 9.07, *p* < 0.001) and exhibited higher receptor levels in the ventral portion (*M* = 1.28, *SD* = 0.2) as opposed to the dorsal (*M* = 1, *SD* = 0.19) and intermediate ones (*M* = 1.1, *SD* = 0.23; Figure 6D).

Receptor expression was of similar level (*F*_(2,57)_ = 2.018, *p* = 0.142) in the dorsal (*M* = 1, *SD* = 0.16), intermediate (*M* = 1.02, *SD* = 0.17) and ventral pyramidal cell layers of the CA1 region (*M* = 1.1, *SD* = 0.19; Figure 6B).

## Discussion

In this study, we report that the D2R expression differs across the dorsal, intermediate and ventral hippocampus of rat. Specifically, we demonstrate that the total D2R levels are the highest in all layers of the ventral DG and CA3 regions, as well as in the dendritic layers of the ventral CA2 and CA1 regions. The dorsal hippocampus shows the overall lowest D2R content, while the intermediate part shows levels that are either in-between the differing levels of the dorsal and ventral poles, or alternatively are equivalent to levels detected in one of the poles. Testing of antibody specificity revealed protein labeling at 55 kDa. This corresponds to the D2L dopamine receptor that is postsynaptic (Lindgren et al., 2003; Gemechu et al., 2018).

Importantly, our study is the first to provide a more complete picture of D2R expression across the hippocampal dorso-ventral axis, as the majority of already published studies focused on brain regions other than the hippocampus (Vincent et al., 1993; Sesack et al., 1994; Yung et al., 1995; Lazarov and Pilgrim, 1997; Jung and Bolam, 2000) or on animal species other than the rat (Khan et al., 1998; Gangarossa et al., 2012; Puighermanal et al., 2015; Wei et al., 2017). Only very few studies report on aspects of the D2R expression in the rat hippocampus. Among them, an immunocytochemical study by Levey et al. (1993) described relatively low D2R immunoreactivity in the hippocampus on sagittal sections from albino rat brains. An earlier autoradiographic study reported the presence of D2R high-density binding sites in the Stratum lacunosum-moleculare of the dorsal CA1 and lower densities in the rest of the rat hippocampus (Charuchinda et al., 1987). The most recent work by Yu et al. (2019) demonstrated cellular expression patterns of D2R in transgenic rat forebrain. They studied D2R positive cells across the transverse axis of only the dorsal hippocampus and reported on D2R expression in the pyramidal cell layer and Stratum radiatum of the CA1–3 regions and the DG, as well as in axon terminals innervating the Stratum lacunosum-moleculare of the CA1 region. With regard to the dorso-ventral distribution of the D2R only one study has addressed this to date. Using a radioligand binding assay, Bruinink and Bischoff, 1993, demonstrated a bimodal distribution from the dorsal to the ventral hippocampus that comprised a high D2R density in one portion of the dorsal part and in the entire ventral part. The results of our current study are in line with this report. We show here that the ventral hippocampus has the highest expression density of D2R. The discrepancy between a bimodal distribution in the aforementioned study and a lack thereof in our case, is probably because in our examination of the D2R expression we used sections from the dorsal part that are very close to the tip of the hippocampus, while in the Bruinink and Bischoff study the dorsal hippocampus was separated into three subdivisions, whereupon the one closest to the intermediate hippocampus showed the high receptor density. Moreover, this study showed that the proportion of the D2R in the high affinity state is higher in the dorsal hippocampus as opposed to the ventral one (Bruinink and Bischoff, 1993). This may have masked differences in receptor distribution, especially considering that the amount of dopaminergic projections reaching the hippocampus is more dense in the ventral portion (Verney et al., 1985). Interestingly, dopamine D1 receptors show a similar pattern of expression along the dorsoventral axis (Dubovyk and Manahan-Vaughan, 2018), with higher levels being apparent in the ventral and intermediate CA1 region compared to the dorsal part. Here, in particular, expression is higher in the ventral and intermediate Stratum lacunosum-moleculare and Stratum radiatum of the CA1 region (Dubovyk and Manahan-Vaughan, 2018).

Studies that scrutinized the binding affinity of D1-like and D2-like receptors (Edelmann and Lessmann, 2018) showed that, at low concentrations, dopamine activates presynaptic D2R leading to a reduction of excitatory responses in the hippocampal CA1 neurons. These findings suggest that during the tonic firing of dopaminergic neurons projecting to the hippocampus, the D2R become activated first, adding to a generalized inhibition of neuronal excitatory postsynaptic currents. This interpretation is consistent with findings, in behaving rats, that intracerebral application of a D2R agonist reduces basal synaptic transmission in the hippocampus (Manahan-Vaughan and Kulla, 2003). These low concentrations of D2R agonist also inhibit vulnerable forms of LTP (Manahan-Vaughan and Kulla, 2003). This process may serve to enhance signal-to-noise ratios and prevent the long-term storage of less salient information.

At higher concentrations, dopamine begins to activate other D2-like receptors followed by D1-like receptors (Gribkoff and Ashe, 1984; Hsu, 1996; van Wieringen et al., 2013). In line with this, high D2R agonist concentrations increase the spontaneous firing rate of dorsal CA1 pyramidal neurons in rat slices (Smiałowski and Bijak, 1987), as well as in the dorsal DG granule cells of freely moving rats (Yanagihashi et al., 1991). Given our finding of an overall dorso-ventral gradient in the D2R expression, it is tempting to assume that the ventral hippocampus may be under a stronger inhibitory control upon tonic dopamine release, but may have an increased neuronal excitability when phasic elevations of dopamine levels occur. This may explain why neuroleptics that antagonize dopamine D2R ameliorate the symptoms of acute, or positive, symptoms of psychosis (Giannini et al., 2000; Dold et al., 2015): by antagonizing the receptor, the balance of activity is restored to one that mediates neuronal inhibition, as opposed to excitation. Our study suggests that these effects may be mediated predominantly by the ventral hippocampus, an interpretation that is supported by studies that suggest that the ventral (and not the dorsal) hippocampus engages predominantly in information processing related to affective state (Fanselow and Dong, 2010).

The involvement of D2R signaling in the modulation of hippocampal synaptic plasticity has been described by several studies. For example, pharmacological blockade of D2R, using a ligand concentration that does not affect basal synaptic transmission, prevents a weak form of long-term potentiation (LTP) in the dorsal DG *in vivo* (Manahan-Vaughan and Kulla, 2003). This study also reported an involvement of the D2R in the regulation of depotentiation, which involves a reversal of recently induced LTP (Manahan-Vaughan and Kulla, 2003). With regard to the CA1 region of hippocampal slices, antagonism of D2R inhibits LTP maintenance but has no effect on the initiation phase (Frey et al., 1989, 1990). Activation of D2-like receptors results in inhibition of long-term depression (LTD) in the CA1 region *in vitro* (Chen et al., 1996). While all of the reported studies on synaptic plasticity have been performed on the dorsal part of the rat hippocampus, only one such study was performed on the ventral part, in this case, of the mouse hippocampus (Rocchetti et al., 2015). Here, the authors reported that both LTP and LTD were severely impaired in the ventral CA1 of hippocampal slices from D2R knockout mice, as well as following the pharmacological blockade of the D2R in naive mice. Moreover, they showed that specific deletion of presynaptic D2R leads to deficits in LTD, but not in LTP. All of these studies show that D2R plays a significant role in the expression of hippocampal synaptic plasticity at both, dorsal and ventral hippocampal parts and, at least, in the DG and CA1 regions.

Mechanistically, the D2R-dependent modulation of synaptic plasticity may occur through GABAergic interneurons, as receptor activation was shown to reduce GABA synthesis in the hippocampus (Steulet et al., 1990), thus allowing for LTP to occur (Wigström and Gustafsson, 1983). However, higher D2R expression in the ventral hippocampus does not seem to be able to fully compensate for differences in LTP between the dorsal and ventral hippocampal parts, as LTP is known to be of lower magnitude in the ventral CA1 compared to its dorsal counterpart (Dubovyk and Manahan-Vaughan, 2018). This difference relates to expression levels of subunits of the NMDA receptor (Dubovyk and Manahan-Vaughan, 2018), but may also relate to differences in D2R expression and/or to the expression of other neurotransmitter receptors. Further studies are required to access the precise contribution of D2R to synaptic plasticity in other CA regions as well as in the intermediate hippocampus.

Functionally, the D2R has been implicated in hippocampus-dependent learning and memory: systemic application of receptor blockers triggers an array of learning and memory deficits (Gasbarri et al., 1993; Sigala et al., 1997; Stuchlik et al., 2007; Rocchetti et al., 2015). Similarly, D2R knockout mice have impaired both, short-term and long-term spatial memory as well as recognition memory (Rocchetti et al., 2015). Interestingly, a direct infusion of a D2R antagonist into the ventral hippocampus of wildtype mice reproduced learning deficits observed in the D2R knockout mice (Rocchetti et al., 2015). The same was found for the rat upon D2R blockade in the ventral hippocampus (Wilkerson and Levin, 1999; Umegaki et al., 2001), suggesting that the systemic effect on spatial learning and memory may, in fact, be mediated by the ventrally expressed D2R. Interestingly, systemic injection of D2R agonist (Imperato et al., 1993, 1994), as well as focal injection into the ventral hippocampus, dose-dependently stimulates acetylcholine release (Umegaki et al., 2001). However, injection of the D2R agonist into the dorsal hippocampus had no such effect on hippocampal acetylcholine release (Day and Fibiger, 1994). Therefore, the involvement of the D2R in memory performance in the ventral hippocampus may happen through the regulation of acetylcholine release.

Accurate spatial navigation is thought to be mediated by place-cell representation of spatial context (Eichenbaum et al., 1999) that is known to differ in the dorsal and ventral hippocampus (Strange et al., 2014). Here, the density of place cells, size of the place fields and their precision are known to gradually change from pole to pole (Jung et al., 1994). Importantly, in D2R knockout mice, the number of place cells is significantly decreased and is accompanied by changes in basic firing properties (intra-field firing rate, spatial tuning and spatial coherence) and reduced stability of place fields (Nguyen et al., 2014). Therefore, the difference in D2R expression between the dorsal and ventral hippocampus that was found in the current work may also account, in part, for dorso-ventral differences in place cells and place fields properties, whereupon the dorsal part encodes high-resolution spatial information for a small environment, while the ventral part processes low-resolution information for a larger environment.

It has been proposed that the hippocampal dorso-ventral axis is functionally segregated on a cognitive level. Whereas the dorsal part is predominantly engaged in visuo-spatial information processing, the ventral part is involved in emotional, motivational and affective responses (Fanselow and Dong, 2010; Strange et al., 2014). The intermediate part may integrate information from both poles (Bast et al., 2009; Fanselow and Dong, 2010; Strange et al., 2014). Interestingly, the ventro-intermediate hippocampal subfields receive a more intense afferent projection form the VTA than does the dorsal subfield (Gasbarri et al., 1994). The higher expression levels of D2R receptors as reported in the present study, along with higher levels of D1DR as reported previously (Dubovyk and Manahan-Vaughan, 2018), suggest that the ventral hippocampus is subjected to quite potent control by the VTA. This indicates in turn, that the ventral part of the hippocampus should be subjected to much closer scrutiny in terms of obtaining an understanding of how the dopaminergic system engages with the hippocampus, and enables hippocampal information encoding and hippocampus-dependent non-spatial behavior.

## Conclusion

Taken together, our study demonstrates that the overall expression of dopaminergic D2R is strikingly different between the ventral pole of the hippocampus and its dorso-intermediate two-thirds of the dorso-ventral axis. In all hippocampal sublayers studied, expression levels were always highest in the ventral compared to the dorsal pole. This difference is likely to contribute to known dorso-ventral differences in synaptic plasticity (Dubovyk and Manahan-Vaughan, 2018) as well as to state-dependent learning and memory.

## Data Availability Statement

The data that support the findings of this study are available from the corresponding author upon reasonable request.

## Ethics Statement

The animal study was reviewed and approved by Landesamt für Arbeitsschutz, Naturschutz, Umweltschutz und Verbraucherschutz, NRW, Germany.

## Author Contributions

The study was designed by DM-V and VD. Experiments were conducted by VD and analyzed by both authors. VD and DM-V wrote the article.

## Conflict of Interest

The authors declare that the research was conducted in the absence of any commercial or financial relationships that could be construed as a potential conflict of interest.
